# Deep vein thrombosis in a patient with Cronkhite-Canada syndrome: a complex case report

**DOI:** 10.1186/s12959-023-00473-8

**Published:** 2023-03-15

**Authors:** Xiao-Kai Feng, Xiao-Fen Chen, Bei-Bei Wang, Zhi-Gang Zeng, Chao Liu, Wei-Hong Sha, Juan Ma

**Affiliations:** 1 Department of Respiratory and Critical Care Medicine, Beijing Institute of Respiratory Medicine and Beijing Chao-Yang Hospital, Capital Medical University, Beijing, China; 2 Department of Gastroenterology and Hepatology, Guangdong Provincial People’s Hospital (Guangdong Academy of Medical Sciences), Southern Medical University, Guangzhou, China; 3 Shantou University Medical College, Shantou, China; 4 Department of Pathology, Guangdong Provincial People’s Hospital (Guangdong Academy of Medical Sciences), Southern Medical University, Guangzhou, China; 5 Diagnosis and Treatment Center of High Altitude Digestive Disease, Xining Second People’s Hospital, Xining, China

**Keywords:** Cronkhite-Canada syndrome, Nephrotic syndrome, Infectious enteritis, Deep vein thrombosis, Methylprednisolone

## Abstract

**Background:**

Cronkhite-Canada syndrome (CCS) is a rare disease characterized by generalized gastrointestinal polyps, ectodermal abnormalities and variable gastrointestinal symptoms. Few cases to date have described complications with deep vein thrombosis (DVT). Here we reported a rare case of CCS concomitant with DVT. The patient’s clinical details, endoscopic findings, safety, and efficacy are reported.

**Case presentation:**

A 58-year-old patient was admitted to our hospital with recurrent diarrhea, overall abnormal appearance, including hyperpigmentation, hair loss and onychodystrophy, and multiple polyps distributed along the gastrointestinal tract except the esophagus. After considerable assessment, the patient was diagnosed with CCS. She was also diagnosed with concurrent DVT, nephrotic syndrome, and infectious enteritis during the course of disease. After treatment with a combination of methylprednisolone, mesalazine, antibiotics, rivaroxaban, and nutritional support during the 24 months of following the patient in this case, the clinical manifestations and endoscopic findings reached complete remission two years after the diagnosis.

**Conclusion:**

To our knowledge, this study is the first case of CCS complicated with DVT reported in China. Although rare, it is important to consider that DVT may occur after CCS and that it is vital to conduct careful follow-up.

## Background

Cronkhite-Canada syndrome (CCS) is a rare disease characterized by generalized gastrointestinal polyps and ectodermal abnormalities including hyperpigmentation, hair loss and onychodystrophy with variable gastrointestinal symptoms [1]. The incidence rate of CCS is approximately 1 case per million persons. Around 500 cases of CCS have been reported to date, with 75% of reports coming from Japan. Variable complications may develop in CCS, including gastrointestinal malignancies, nephrotic syndrome, sepsis, deep vein thrombosis (DVT), myelodysplastic syndrome, aplastic anemia, and acute encephalopathy syndrome [2–6]. The mortality rate of CCS is as high as up to 50% which may be attributed to the diverse complications mentioned or the lack of a well-recognized effective treatment regimens for the disease [7].

Furthermore, DVT may play an important role in the prognosis of CCS, impacting both the disease itself and its treatment. Therefore, proper and timely evaluations are critical in throughout the treatment process. Here, we reported the case of CCS concomitant with DVT that achieved an exceptional therapeutic effect.

## Case presentation

On August 16, 2018, a 58-year-old female was admitted to our hospital, complaining of a 2-month history of anorexia, abdominal distention exacerbated by food intake, watery diarrhea, malaise and 8-kg weight loss. She denied the diarrhea was related to the ingestion of lactose, gluten-containing food, or emotional changes, but mentioned intake of Chinese medicine before onset.

**Fig. 1:**
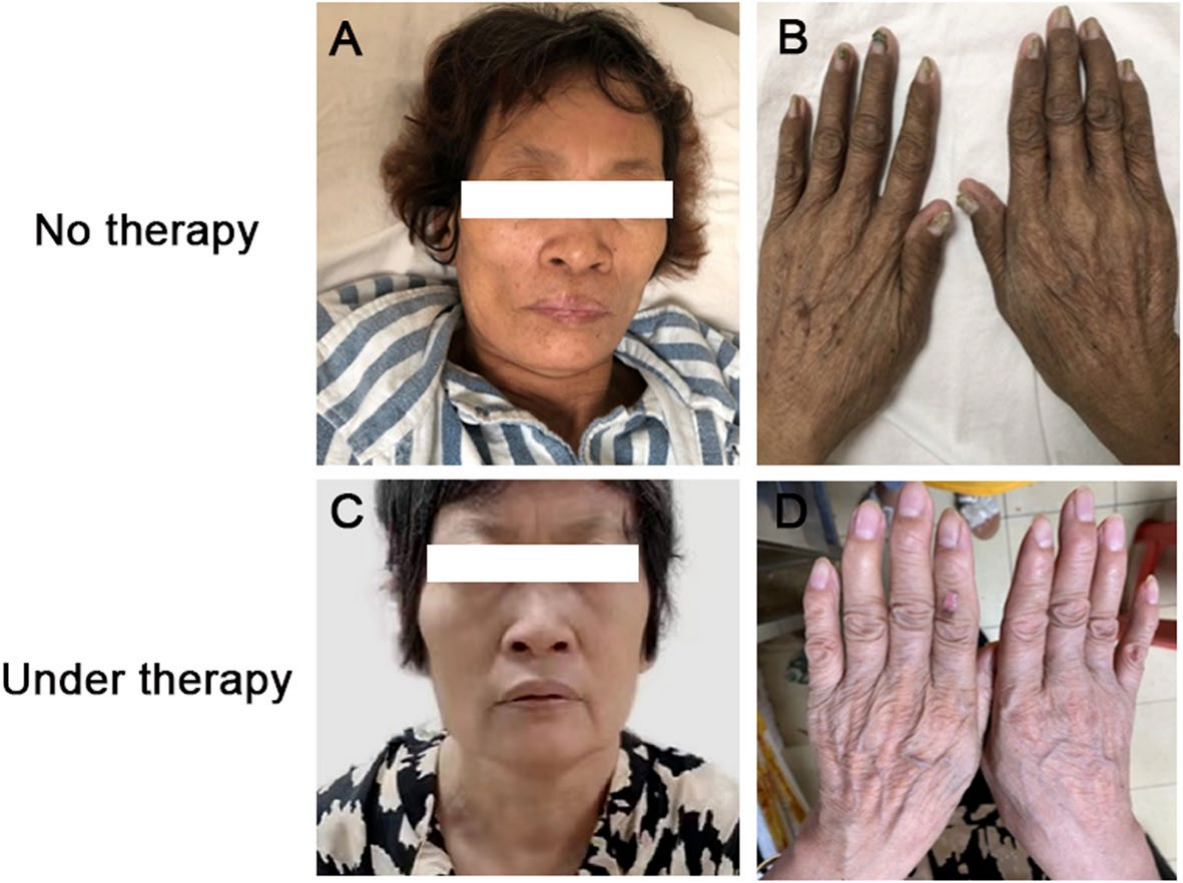
Typical appearance changes of the patient before therapy (**A-B**) and after methylprednisolone-based comprehensive therapy (**C-D**)

Physical examination revealed the patient was malnourished, with a body mass index (BMI) of 16.02 kg/m^2^, and the appearance of a chronic illness, including sparse hair, black-brown pigmentation of palms and feet, and dystrophic nails (Fig. 1A and B). Laboratory tests showed decreased white blood cells (3.11 × 10^9^/L), complement C3 (497.9 mg/L), and albumin (37.6 g/L). Other blood parameters, including liver, renal, thyroid function, and tumor markers, were normal.

Autoimmune assessments indicated a slight abnormality in patient’s anti-ds-DNA (20.8 IU/mL) and antinuclear antibody (titer 1:80, discrete speckle pattern), with all other tests within normal range, including antinuclear antibody profile, indicators for screening early rheumatoid disease, vasculitis, antiphospholipid-antibody syndrome, and autoimmune hepatitis.

Furthermore, the patient’s stool sample reviewed no occult blood, ova, or parasites. Gastroduodenal endoscopy revealed erosive gastritis and duodenitis with swollen and protruding gastric mucosa. Infection with *H. pylori* was excluded from the diagnosis by the rapid urease test and hematoxylin–eosin staining. the patient’s colonoscopy revealed edematous terminal ileum mucosa with inflammation, and multiple polypoid eminences were removed by endoscopic electronic resection and clamping. A video capsule and double-balloon endoscopy revealed atrophy with a mosaic and serrated appearance.

Malignancy was excluded by PET-CT, and toxicological testing was negative for heavy metal intoxication from herbal medicine. The patient denied any family history of gastrointestinal polyposis or malignancy, and she had no mutations in cancer-related genes, including *APC, ATN2, BARD1, BRCA1, MSH,* or and *MUTYH* tested by next generation sequencing.

**Fig. 2:**
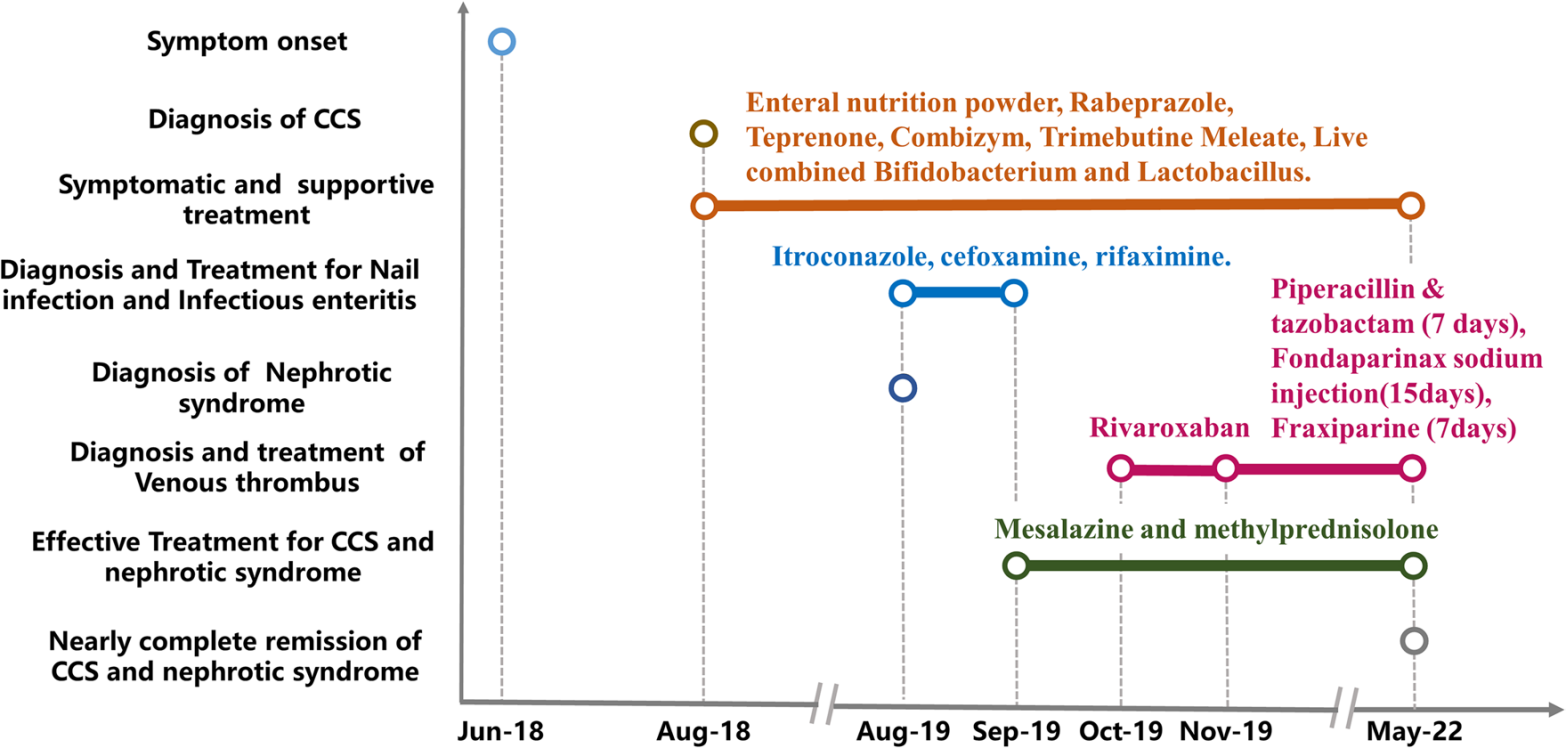
Time-dependent figure of disease progression and treatment

CCS was given the clinical manifestation of gastrointestinal and ectodermal symptoms, massive polyps throughout the gastrointestinal tract, and the special pathological features. The patients was prescribed 3000 mg/day mesalazine with symptomatic and supportive treatment, including nutritional support (enteral nutrition powder), gastric mucosa protection (rabeprazole & teprenone), digestive enzyme supplementation (Combizym), gastrointestinal peristalsis regulation (trimebutine meleate) and intestinal flora modulation (live combined *Bifidobacterium* and *Lactobacillus*) (Fig. 2). The patient saw improvement in gastrointestinal symptoms and body weight following treatment.

**Fig. 3:**
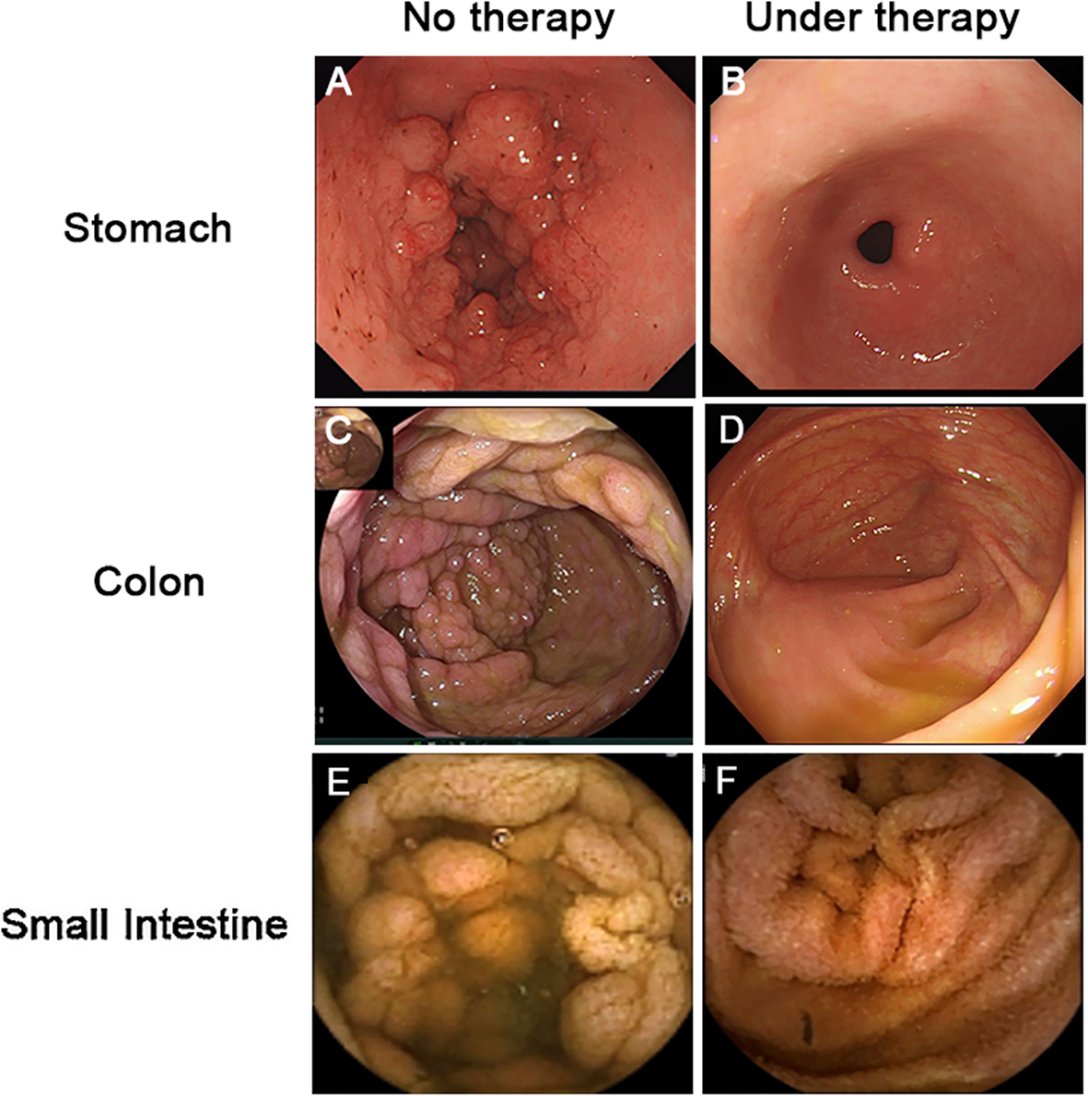
Representative images of gastroscopy, colonoscopy and video capsule endoscopy. Before effective treatment, gastric antrum mucosa was swollen with scattered punctate pigmentation, dense granular and nodular uplift (**A**), a laterally developing tumor-like protrusion in the cecum, multiple polyps along the colon (**C**), and edema and atrophied of mucosa and villi of small intestine with mosaic and sawtooth signs and diffuse polypoid lesions (**E**). After methylprednisolone-based comprehensive therapy, both gastric and colonic mucosa became smooth and all polyps subsided (**B**, **D**), edema and atrophied mucosa and villi were reduced, and small intestine polypoid lesions disappeared (**F**)

However, 1 year later, the patient was re-admitted to the hospital due to recurrent diarrhea, exaggerated weight loss, fever, lower extremity edema and foamy urine, and additional symptoms. A repeat gastroscopy and colonoscopy revealed aggravation of the mucosa edema and diffuse eruption of polyps in the stomach and colon (Fig. 3A and C). Additionally the patient’s video capsule endoscopy indicated atrophy with a nodular and serrated appearance of the small intestinal mucosa and diffuse polypoid lesions (Fig. 3E).

**Fig. 4:**
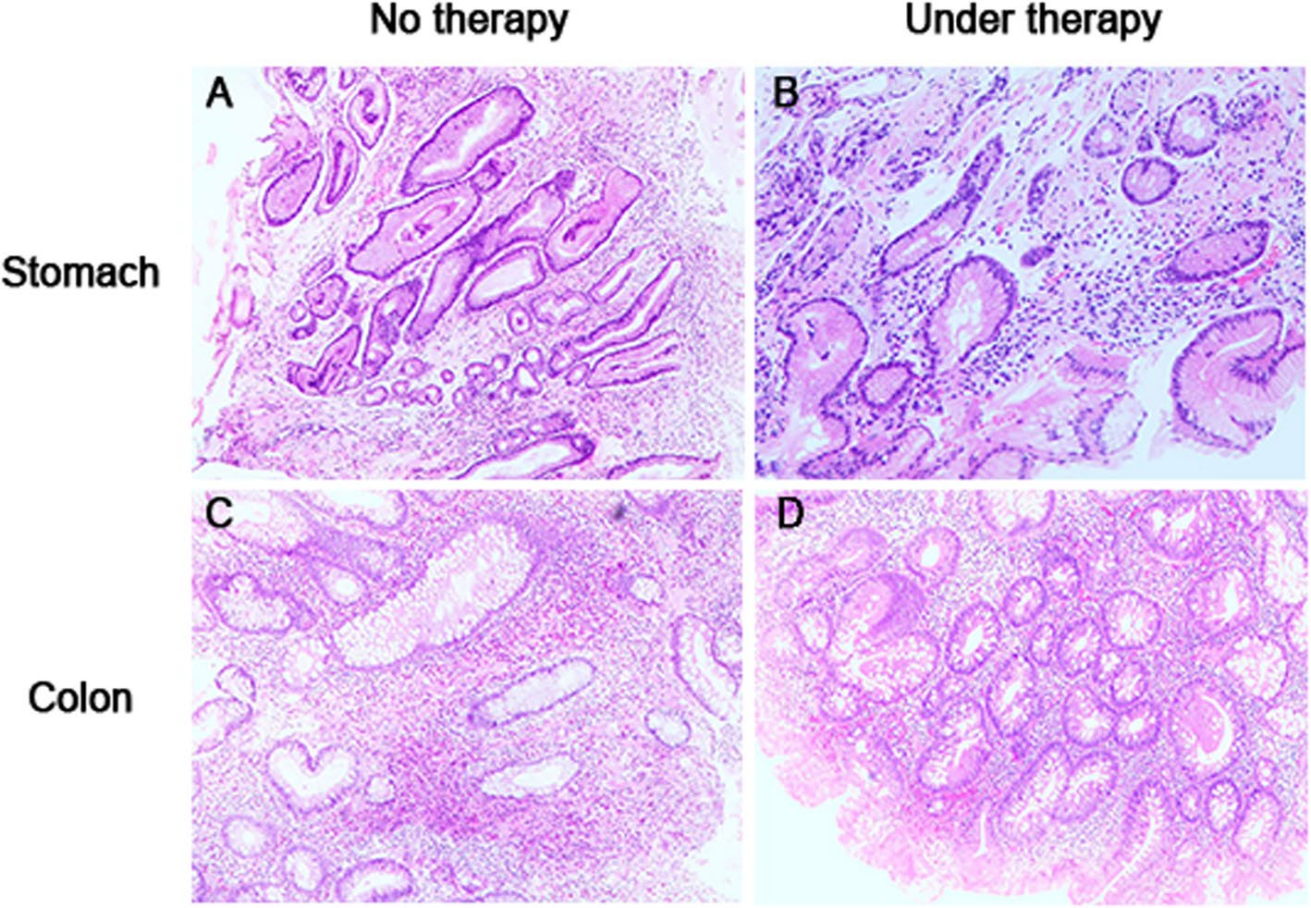
Representative histological photos of endoscopic biopsy specimens taken from the stomach and colon (Hematoxylin–eosin staining, original magnification × 100). Before effective treatment, there was chronic inflammation, extensive interstitial edema, gland expansion and hyperplastic polyps in stomach were present (**A**), inflammatory cell infiltration in the colonic mucosa was present, and tubular adenomas were widely distributed from the cecum to the rectum with or without low-grade intraepithelial neoplasia (**C**). After the methylprednisolone-based comprehensive treatment, only mild chronic inflammation was present in the gastric mucosa (**B**), and scattered tubular adenoma was present in the colon (**D**)

Further histological examination revealed infiltration of mixed inflammatory cells, excessive stromal edema, sparse and shrinking villi and crypts, hyperplastic polyps in stomach, and tubular adenoma widespread from the cecum to the rectum (Fig. 4A and C). Nail atrophy worsened with exudation, and we then diagnosed the patient with a *Candida glabrata* infection*.* The microbiological culture of the stool further confirmed a salmonella infection. Laboratory tests for the inflammatory-related cytokines indicated normal levels of interleukin-6 (4.1 pg/mL) and ceruloplasmin (281 mg/L), with a decreased level of α1-acidglycoprotein(0.39 g/L). Additional laboratory tests showed hypoproteinemia (serum albumin 19.84 g/L) and massive proteinuria (24-h urine albumin 6.0 g/L). The patient’s anti-PLAR2 antibody was negative, and blood tests for hepatitis B (HBV), hepatitis C (HCV), human immunodeficiency virus (HIV), and syphilis infections were negative.

**Fig. 5:**
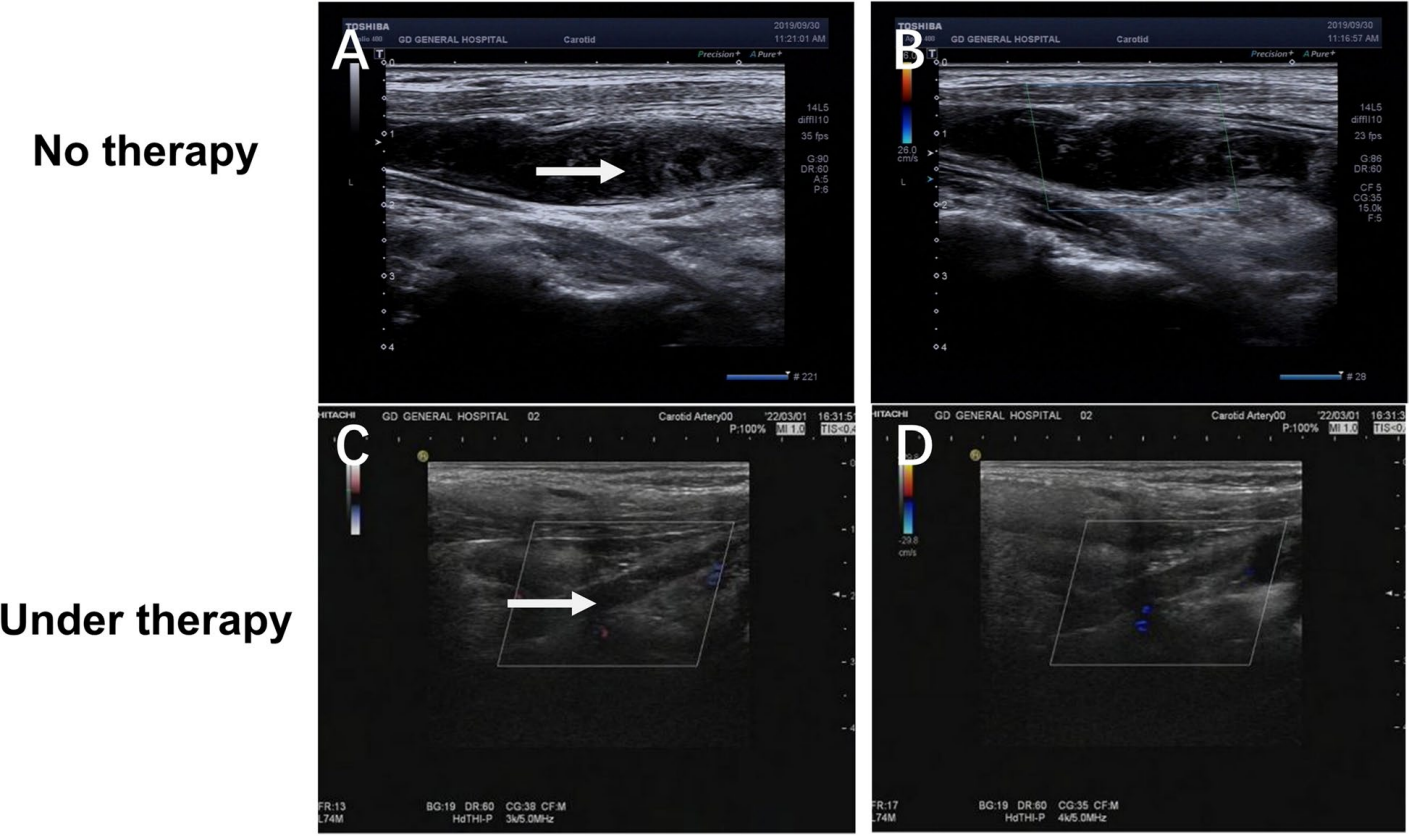
An ultrasound examination of the neck demonstrated low-density thrombus (white arrow) in the left internal jugular vein after catheterization of PICC (**A**) and Color Doppler imaging confirmed the blockage of blood flow (**B**). After adequate dose and duration of anti-coagulation, surveillance ultrasound detected that the thrombus (white arrow) remained stable in size with increased density in the left internal jugular vein (**C**), with no recovery of blood flow in Color Doppler image (**D**)

**Fig. 6:**
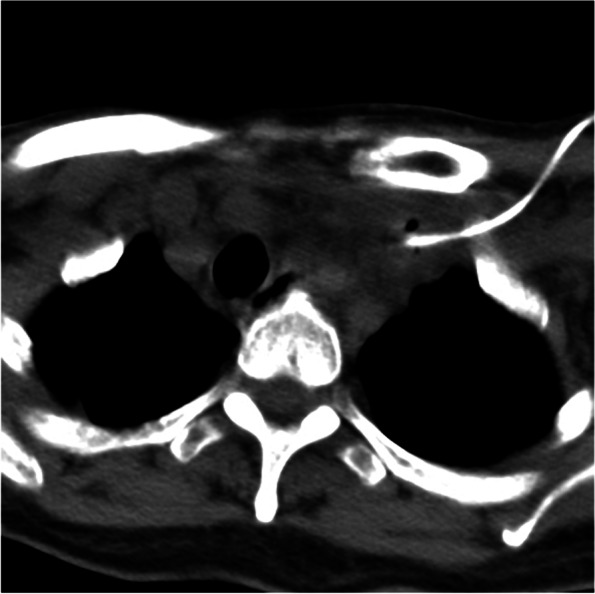
CT scanning detected the PICC in the left subclavian vein and left internal jugular vein with thrombus formation. A few gas bubbles were seen along the left internal jugular vein Excessive exudation and inflammatory changes were seen in the interstitial space around the venous thrombus.

Given the patient’s hypoalbuminemia and proteinuria results, nephrotic syndrome diagnosis was certain. Because the patient had severe fluid loss and malnutrition due to watery diarrhea, a central venous catheter (CVC) placed in the left internal jugular vein to facilitate fluid infusion and parental nutrition. However, 6 days following insertion of CVC, the patient complained about neck pain and skin swelling. No coagulation function and serum protein electrophoresis abnormalities were found. However, Color-Doppler ultrasound detected thrombus formation of the left internal jugular vein involving the proximal end of left subclavian vein(Fig. 5A and B), A CT of the neck confirmed the findings and found excessive exudation in the interstitial space with inflammatory changes in the surrounding tissue around the venous thrombus (Fig. 6). Before and after the formation of the venous thrombus, the platelet level increased from 249 × 10^9/L to 316 × 10^9/L, international normalized ratio (INR) decreased from 1.03 to 0.94, and fibrinogen increased from 4.93 g/L to 6.15 g/L. However, there prothrombin time (12.7 s), activated partial thromboplastin time (43.3 s), thrombin time (14.2 s), and level of D-dimer (1010 ng/mL) did not change significantly. The CVC was immediately removed to avoid its influence on the DVT, and the patient was treated with empirical use of piperacillin and tazobactam (4.5 g, iv, q8h) and a fondaparinux sodium injection (0.5 ml, qd) to prevent further thrombosis. The patient had a relief of neck pain and skin swelling after treatment but relapsed later, therefore, the patient was given low molecular weight heparin (LMWH 0.4 ml, subcutaneous injection, q12h) and discharged with rivaroxaban (10 mg, qd).

The patient was diagnosed as CCS complicated with infectious enteritis, nephrotic syndrome and jugular vein thrombosis. A comprehensive therapy consisting of methylprednisolone, mesalazine, anti-infection, anticoagulant treatment, and auxiliary nutritional support were provided. Two years later, the patient reached complete remission of gastrointestinal symptoms and was well-nourished with a BMI of 24.03 kg/m^2^.She had grown back a full head of glossy, black hair, had complete disappearance of pigmentation on her hands and feet, and regrowth of healthy nails (Fig. 1C and D). Hypoproteinemia and proteinuria returned to normal (serum albumin 41.7 g/L). On March 1, 2022, the patient received gastroscopy and colonoscopy, which showed a significant reduction in the number of polyps(Fig. 3B, D, and F), and pathological examination revealed only mild inflammation throughout the gastrointestinal tract and regeneration of the intestinal villi and crypts (Fig. 4B and D). The patient had no recurrence of neck pain or skin swelling. Surveillance ultrasound detected that the left internal jugular vein thrombosis still existed but remained stable in size with organization (Fig. 5C and D). The patient had regular followed-up appointments to monitor her long-term prognosis, including maintenance of remission, recurrence of CCS, and potential canceration of colonic polyps.

## Discussion and conclusions

The patient in the case developed DVT 6 days after insertion of a CVC during the re-hospitalization for exaggeration of previous CCS and onset of new symptoms 1 year after diagnosis. We present here a paradigmatic case with complex interplay of risk factors that can lead to thrombosis in a CCS patient. This case was suggestive of a CCS -associated DVT that raised questions on how to properly manage DVT in such an underexplored situations.

DVT in this patient may have resulted from several factors. First, because autoimmune dysfunction is the most well-known and widely accepted etiological cause for CCS, we assessed a number of autoimmune indicators associated with her symptoms. In this patient’s case, all the common autoimmune disease-related factors were within normal range, making rheumatic immune diseases less possible. The relationship between inflammation and thrombosis has been identified in various clinical scenarios where the inflammatory process and coagulation abnormalities are clearly interlinked. A case report by Sampson et al. described a patient suffering from repeated occurrences of pulmonary and distal arterial thrombosis with every attempt to stop anticoagulation, in spite of adequate duration of anticoagulation [8]. Elevated levels of fibrinogen and Factor VIII coagulant activity play an important role in the coagulation abnormalities, which resulted in this CSS patient suffering from repeated occurrences of pulmonary and distal arterial thrombosis [8].

Furthermore, several cases of elevated antinuclear antibody levels have been found in thrombosis-complicated CCS events, prompting the field to conclude that the development of thrombosis likely has an autoimmune etiology as well [8, 9]. Accumulating evidence supports that DVT formation is not restricted to coagulation system but it is also closely related to inflammation [10]. Inflammatory related cytokines, chemokines, and leukocytes likely contribute to the formation of DVT [11]. A possible mechanism of a DVT is the inflammation of the vessel wall via elevated C-reactive protein (CRP), IL-6, IL-8, and tumor necrosis factor, which induce expression of tissue factor, promoting a pro-coagulant state, leading to thrombus formation in an intact vein [10–12]. The patient in our case in a hypercoagulabale state with an elevated level of fibrinogen and D-dimer, suggesting the formation of venous thrombosis. Prior to the thrombotic event, she was diagnosed with infectious enteritis caused by salmonella and nephrotic syndrome, both of which may have irritated her inflammatory state.

Additionally, pathophysiological mechanisms of thrombosis can be explained by the Virchow’s triad: stasis, vascular wall damage or dysfunction, and hypercoagulability. Once the Virchow’s triad is disturbed, the natural anticoagulant properties of the endothelium will be downregulated and then induce a hypercoagulable state. Activated leukocytes will express the procoagulant tissue factor to initiate the extrinsic pathway of the coagulation cascade, leading to formation of thrombus comprising fibrin, red blood cells, and platelets [13]. CVC insertion is required for long-term parental nutrition. In this case, our patient with chronic intestinal failure relied on parenteral nutrition to preserve life via the CVC. CVC insertion is frequently used for short-term venous access. However, previous research suggests that rates of midline DVT can range from 1 to 4% [14, 15]. At the time of the line placement, the vein can become injured and thrombogenic due to the release of tissue factor; and the catheter creates a turbulent blood flow, thereby activating the coagulation cascade. This activity may help the medicines in catheter to play a role in the thrombosis [16]. In our case, the CVC (DIALL, China) was 2.4 mm in diameter and was inserted via the left sub-clavicular vein due to the difficulty from the right side. This insertion made the patient vulnerable to the thrombus formation.

Moreover, activated leukocytes will express the procoagulant tissue factor to initiate the extrinsic pathway coagulation cascade pathway, leading to the thrombus comprising fibrin formation, red blood cells, and platelets [13]. In our CCS patient case, venous thrombus formation in the left internal jugular vein and subclavian veins was formed where the CVC was localized. Prior to this thrombotic event, our patient was diagnosed with infectious enteritis caused by salmonella and nephrotic syndrome. Thromboembolism is also considered to be the most common, major complication of nephrotic syndrome [17–20]. Patients with CCS are usually accompanied by loss of protein from the gastrointestinal(GI) tract and hypoalbuminemia. The comorbidity of nephrotic syndrome in our patient case impacted the deterioration in their albuminemia. Therefore, the risk of a thromboembolic event dramatically increased in this case as albumin has an anticoagulant and antiplatelet aggregation action.

Finally, glucocorticosteroids are a routine therapeutic for the treatment of CCS. However, they are also known to induce hypercoagulability, increase plasma fibrinogen levels, and decrease tissue plasminogen activator activity and prostacyclin synthesis [21].

Our case suggests that there is a possible protective effect of prophylaxis for DVT in CCS patients at higher risk. This also suggests that for CCS, thromboprophylaxis strategies should fully be strengthened, particularly in the presence of CVC, infection, hypoalbuminemia, oral glucocorticoids, and nephrotic syndrome. Although these findings are not surprising, given that the CCS patient here presented at high risk for DVT, our case raised the question of screening for DVT, risk stratification, and potential DVT prophylaxis to improve outcomes in patient with CCS. Further, since DVT has no specific clinical manifestations, it can be easily misdiagnosed at the initial stage, and a combination of these diseases cannot be ruled out. These results can make it difficult to diagnose DVT and treat quickly. Therefore, more attention should be paid to the high-risk DVT in CCS patients and DVT prophylaxis.

In conclusion, though CCS is rare, DVT occur in these patients and is critical to treat appropriately. This rare case report aids in our understanding of the disease and its complications’ features. The risk of thromboembolism during CCS is high and should be taken seriously. Here we discuss the pathophysiological mechanism of the development of a thromboembolic event in CCS and the proper assessment and treatment to achieve the best outcomes. We found that CCS is prone to thromboembolic episodes due to speculated autoimmunity of CCS, complications of CCS (nephrotic syndrome and hypoalbuminemia), and the insertion of CVC for parental nutrition and medications. However, the frequency of DVT in CCS has not yet been determined, and the difference in the risk of DVT in patients with or without CCS remains unknown. This makes our case unique, and we hope it motivates practitioners to implement early screening for DVT in patients with CCS and emphasizes the importance of prophylaxis. Although rare, it is important to keep in mind that DVT may occur after CCS, and careful follow-up is important after diagnosis.

## Data Availability

The datasets obtained and analyzed in the current study are available from the corresponding author upon reasonable request.

## Abbreviations

CCS: Cronkhite-Canada syndrome
DVT: Deep vein thrombosis
BMI: Body mass index
PICC: Peripheral inserted central catheter
CT: Computer Tomography
LMWH: Low molecular weight heparin
GI: Gastrointestinal
